# Prediction models for early detection and diagnosis of lung cancer in people who have never smoked: a systematic review and critical appraisal

**DOI:** 10.1007/s10552-026-02173-w

**Published:** 2026-05-19

**Authors:** Judith Burchardt, Katherine Stokes, Yohance Victory, Weiqi Liao

**Affiliations:** 1 Department of Public Health and Policy, London School of Hygiene and Tropical Medicine, 15-17 Tavistock Place, London, WC1H 9SH UK; 2 Department of Psychology, University of Warwick, Coventry, UK; 3 University of Bristol, Bristol, UK; 4 Division of Cardiovascular Sciences, School of Medical Sciences, Cardiovascular Sciences (Clinical Sciences Wing), Glenfield Hospital, University of Leicester, Leicester, LE3 9QP UK

**Keywords:** Lung cancer, Never smoker, Non-smoker, Systematic review, Critical appraisal, Risk prediction model, Early detection and diagnosis

## Abstract

**Background:**

Never-smokers can develop lung cancer, but this possibility is often overlooked by patients and physicians. We conducted a systematic review to identify existing prediction models that could be implemented in primary care or at a population level to facilitate early detection and diagnosis of lung cancer in never-smokers.

**Methods:**

This study was registered on PROSPERO (ref: CRD42023374471). We systematically searched literature on the Medline, Embase, PsycINFO, and CINAHL databases published before 22 January 2025, with additional hand searching. Primary care or population-level data from subjects including never-smokers had to be used for model derivation. Studies involving specialized tests (radiological or genetic) were excluded. We used CHARMS to guide data extraction and critical appraisal, the TRIPOD statement for model evaluation, and PROBAST for risk of bias assessment.

**Results:**

Among 2,431 studies retrieved, 31 models were included. Eight models were developed exclusively for never-smokers, but none were at low risk of bias. Among 23 models derived from never- and ever-smokers, five were at low risk of bias. Two were diagnostic models with 1–2 years prediction horizons, and three were prognostic models with 2–10 years prediction horizons. Methodological issues from the included studies were identified, analyzed, and discussed.

**Conclusion:**

This systematic review critically appraises and summarizes key information from currently available prediction models for lung cancer in never-smokers. The findings can inform future research to improve care and services for this underserved population.

**Supplementary Information:**

The online version contains supplementary material available at 10.1007/s10552-026-02173-w.

## Introduction

Lung cancer is both the most frequently diagnosed cancer and the leading contributor to cancer death worldwide, accounting for 12% of incident cancers and 19% of cancer deaths [1]. 78% of men and 66% of women with lung cancer were diagnosed at advanced stages (5-year survival 15% for stage III, 5% for stage IV) in the UK in 2021 (the most recently available cancer statistics) [2, 3]. Lung cancer has a sojourn time of 3–6 years before it presents clinically [4]. A meta-analysis of nine trials showed low-dose computed tomography (LDCT) screening of smokers reduced lung cancer mortality by 16% (95% confidence interval, 95% CI 9–24%) and all-cause mortality by 3% (95% CI 0–6%)[5]. LDCT screening has been established in the United States, Australia, Canada, Croatia, Poland, South Korea, and Taiwan [6–8], and is being rolled out in England for ever-smokers aged 55–74 years [9] with either a five-year lung cancer risk ≥ 2.5% using the Liverpool Lung Project (LLP _v2_)[10] model or a six-year lung cancer risk ≥ 1.51% using the Prostate, Lung, Colorectal, and Ovarian Screening trial (PLCO _M2012_)[11] model. Smoking prevalence has been falling in wealthy countries since 1956, when it was identified as an important cause of lung cancer [12]. However, the proportion of lung cancer patients who are never-smokers has increased since this time [13–16]. Currently, 19% in the US, 21% in the UK, 43% of Chinese male, and 87% of Chinese female lung cancer cases are not attributable to smoking [17–20]. Despite this, little attention has been paid to lung cancer in never-smokers [21].

The United States Centres for Disease Control (CDC) defines never-smokers as those who have never smoked or smoked fewer than 100 cigarettes in their lifetime [22]. “Never-smokers” and “non-smokers” (i.e., people with no smoking history) are used synonymously in the literature, with ex-smokers (people with a previous smoking history) being categorized separately. The International Association for the Study of Lung Cancer (IASLC) published a language guide [23] in September 2025, trying to end stigma for the word “smoker” (person who smokes). Their recommended phrases are in parentheses in this paragraph. As the terms “smokers,” “ex-smokers,” “non-smokers,” etc. have been widely used by the research community, to improve readability of this paper, we use “never-smokers” and “ever-smokers” (people with an ever-smoking history, including current and ex-smokers) without judgment or stigmatization of any individuals or groups.

Age-adjusted incidence of lung cancer in self-reported never-smokers in the UK was estimated to be 16/100,000 (95% CI 15–18) person-years in women and 12/100,000 (95% CI 10–13) in men in 2018 [24]. Lung cancer in never-smokers is more common in Asian than in Caucasian people [25, 26] with an adjusted hazard ratio of 2.8 (95% CI 1.6–4.9) in an Australian cohort study [27], and may be more common in females than in males [25, 28, 29].

Lung cancer screening has not been offered to never-smokers in any Western country. It is offered in East Asia [30–32], but may cause overdiagnosis [33–35]. Delayed diagnosis is common as neither patients nor their doctors expect never-smokers to develop lung cancer [36, 37]. Never-smokers with lung cancer are younger and have fewer symptoms and comorbidities than smokers [38], making early diagnosis more challenging. Lung cancer in never-smokers differs from that in ever-smokers, as it is marked by different mutational patterns, histologically is predominantly adenocarcinoma, and has a better prognosis [38, 39]. If considered a distinct entity, lung cancer in never-smokers is the fifth most common cause of cancer-related death globally [39].

To promote early detection and diagnosis of lung cancer in never-smokers, we conducted a systematic review to identify, summarize, and critically appraise published models predicting incident lung cancer in never-smokers, which could be applied in primary care or at a population scale. We comprehensively report model characteristics, including study populations, study designs, predictors, prediction horizons, and model performance statistics. We did not perform a meta-analysis on risk factors, as our primary aim was to summarize and critically appraise currently available models, to enable researchers to select and apply appropriate models in their local populations or to develop and validate new prediction models based on the published models.

## Methods

We registered our systematic review on PROSPERO (ref: CRD42023374471) and followed the PRISMA (Preferred Reporting Items for Systematic Reviews and Meta-Analyses) statement [40] to conduct this study (checklist in Tables S2).

### Literature search

**Fig. 1:**
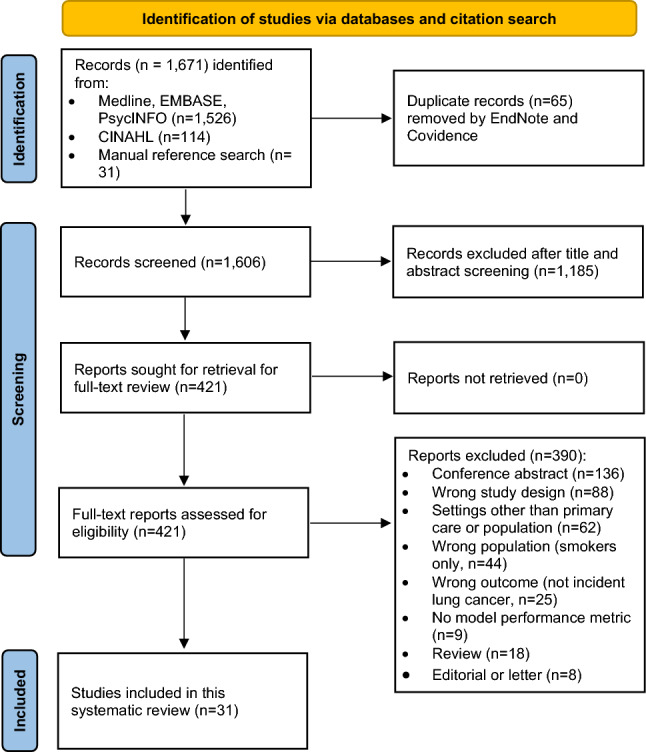
The PRISMA flow diagram for this systematic review

We systematically searched literature from Medline, Embase, PsycINFO, and CINAHL (Cumulative Index to Nursing and Allied Health Literature) databases from inception to 22 January 2025 to capture studies developing and/or validating prediction models for incident diagnosis of lung cancer in never-smokers. We consulted a senior outreach librarian (the University of Oxford Bodleian Health Care Libraries) and iterated our search strategy. We included key terms of “lung cancer,” “non/never-smokers,” and “risk prediction model,” considered relevant derivative words (for example, smoke, smoking, smokers; risk prediction model, risk assessment tool, etc.) with wild cards (*) in our search strategy. Medline, Embase, and PsycINFO were integrated into one platform. The terms were searched in the following nine fields, ab (Abstract), hw (Heading Word), kf (Keyword Heading Word), kw (Keyword Heading), mh (MeSH), ot (Original Title), sh (Subject Headings), ti (Title), and tw (Text Word). We performed the initial literature search (July 2023) and updated it on 22 January 2025 when we wrote up this paper. Table S1 reports the full final strategy. Additionally, we hand-searched references for potentially eligible articles. We used EndNote to manage retrieved references and Covidence for the screening process. Figure 1 presents the PRISMA flow diagram for this systematic review.

### Eligibility criteria

We included models that could be applied to never-smokers from the general or primary care population and excluded models only applicable to ever-smokers. Models using results from secondary care investigations as predictors, such as CT scans or genetic tests, were excluded. We included the outcome of incident diagnosis of lung cancer and excluded mortality/death, as the purpose of this review was to detect and diagnose lung cancer early in never-smokers in the primary care setting or at a population level. We included studies reporting the development of new multivariable models and updates or external validations of existing models. Updating of models may range from simple adjustment of the baseline risk/hazard or additional adjustment of predictors’ coefficients by using the same or different variables to re-estimate predictors’ coefficients, to adding new predictors or removing existing predictors from the original model. External validation studies aim to assess predictive performance of an existing model in an independent population. We included external validation studies that explicitly estimated and presented a measure of the model’s performance and excluded those which did not report these. We included prediction models using case–control or cohort study designs and excluded cross-sectional studies, because prediction models estimate probabilities of a certain outcome for an individual patient over a specified time horizon. Cross-sectional studies measure predictors and the outcome concurrently. Eligible articles were original research and studied humans. We included papers written in any language, using machine translation into English if needed. Table 1 reports the PICOTS (Population, Intervention, Control, Outcome, Timing, Setting) components for this review.

**Table 1:** Inclusion and exclusion criteria for systematic review in the PICOTS framework [83]

Criteria	Inclusion criteria	Exclusion criteria
Population	General population or primary care population who never smoked or stratified based on smoking history as never- or ever-smokers	Non-human or animal samples
Index model (Intervention)	1. Models were developed exclusively for never-smokers, or 2. Models were developed including never-smokers and ever-smokers	Lung lesions or nodules histopathology, genetics, or images
Comparator	NA	NA
Outcomes of interest	Incident diagnosis of lung cancer	Other outcomes such as death
Timing (prediction horizon)	Diagnostic and prognostic models with a predictive horizon for 1—10 years With model performance measures, including discrimination (AUC, concordance c-statistic), calibration (O/E ratio, Brier score), and net benefit (if reported)	Studies did not assess the performance of prediction models
Intended Setting	The models can be applied in the primary care setting or at the population level	Specialized parameters such as genetics or radiology
Type of study design	Any type of studies for prediction models, including randomized controlled trials, prospective/retrospective cohort studies, (nested) case control studies	
Year of publication	Until 22 January 2025	
Publication type	Published, peer-reviewed original research article	Conference abstract, editorials, commentary, letters, expert opinion
Language	All languages	

*AUC* area under the curve, *NA* not applicable, *O*/*E* observed/expected

### Double screening of literature

We imported the references from Endnote and used Covidence (an online tool for systematic review, https://www.covidence.org/) to streamline the review process. Two reviewers independently screened titles and abstracts at the first stage. If their decisions differed, a third reviewer resolved the conflicts and discussed with the two reviewers when necessary. The same process was repeated for full-text review, at which stage the reason for exclusion was documented and reported in the PRISMA flowchart (Fig. 1).

### Data extraction and critical appraisal

One author (WL) constructed a standardized form (Table 2) following the recommendations in the CHARMS (Critical Appraisal and Data Extraction for Systematic Reviews of Prediction Modelling Studies) checklist [41] for data extraction and critical appraisal of the included prediction models. For all eligible studies, we extracted information on the first author, model name, year, and journal of publication. Two authors extracted study design, study population, geographical location, prediction horizon, modelling method (e.g., logistic regression, Cox regression, machine learning methods, etc.), method of internal validation, numbers of never-smokers, and lung cancer cases (events), predictors in the final model, handling of missing data, and measures of model performance (discrimination, calibration, and clinical utility). Data extraction was reviewed by two other authors for quality assurance. If papers presented a model for never-smokers and an additional model for the general population including both ever- and never-smokers, then only the never-smoker model was included, as this was most relevant to our research aim. Potential measures of classification were sensitivity, specificity, positive, and negative predictive values, of discrimination were C statistics, area under the curve (AUC), and D statistics; and for calibration included calibration plots, calibration-in-the-large, calibration slopes, Hosmer–Lemeshow tests, Harrell’s E statistics, and calibration tests [42]. We followed recommendations from the TRIPOD (Transparent Reporting of a multivariable prediction model for Individual Prognosis Or Diagnosis) statement [43] to evaluate the models.

**Table 2:**
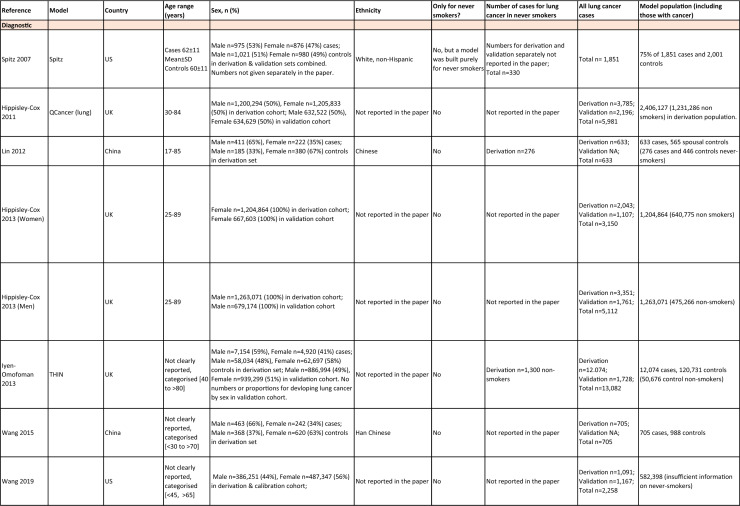

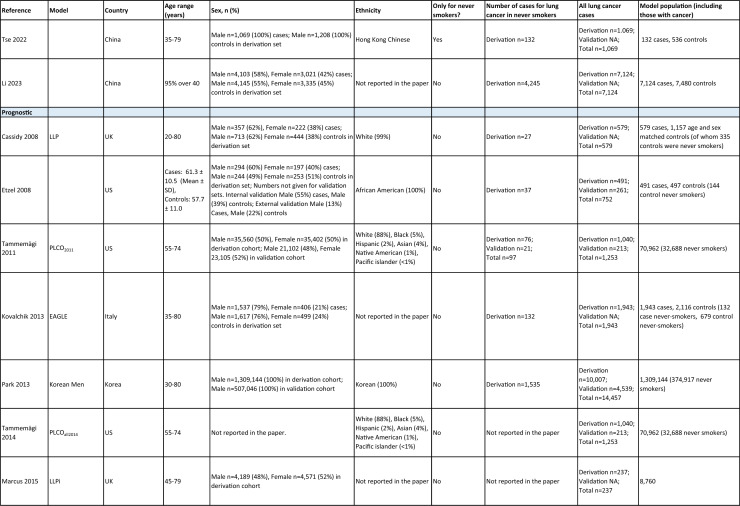

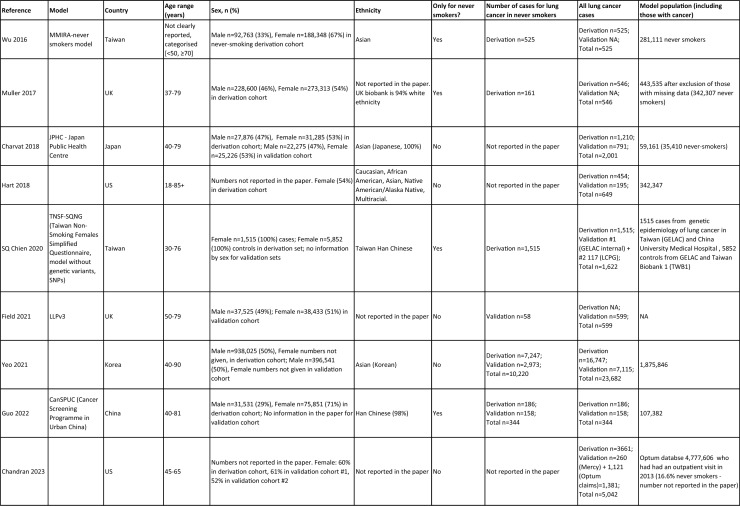

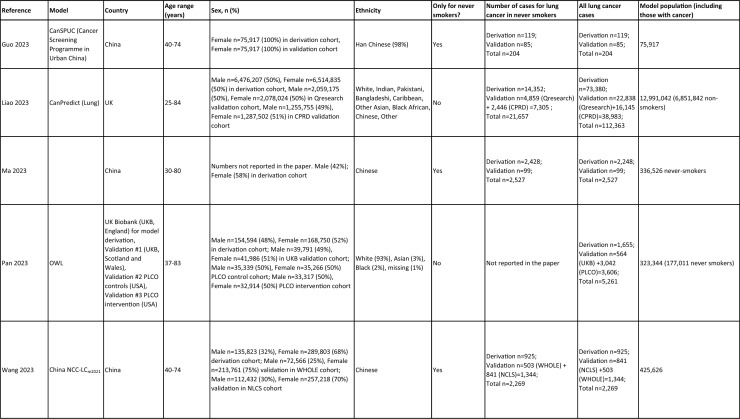

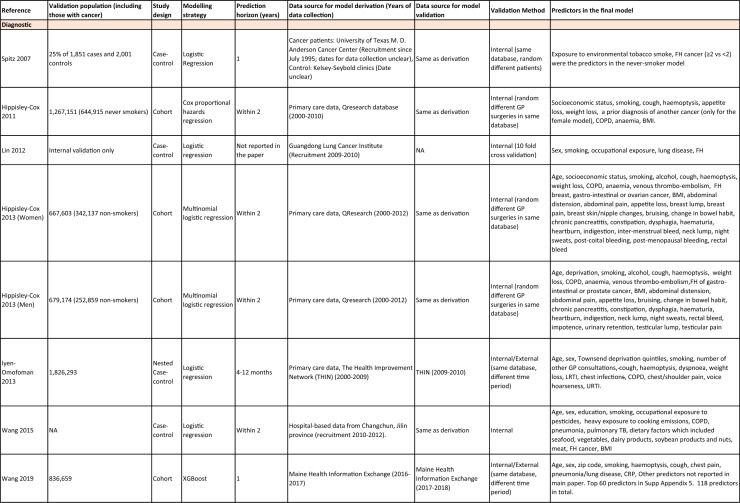

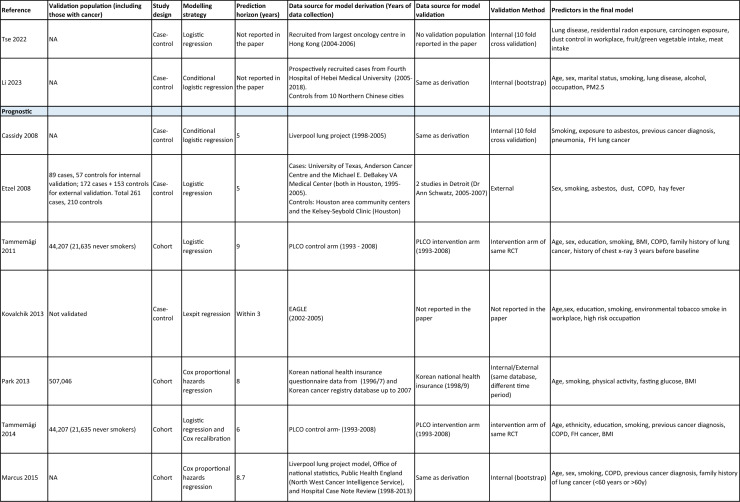

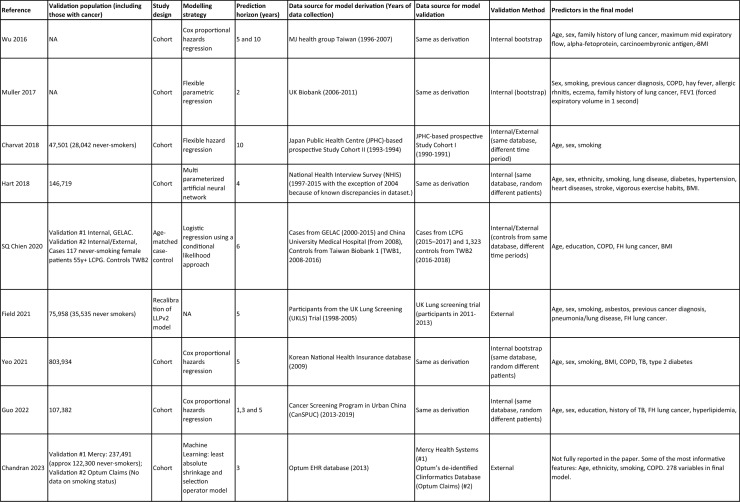

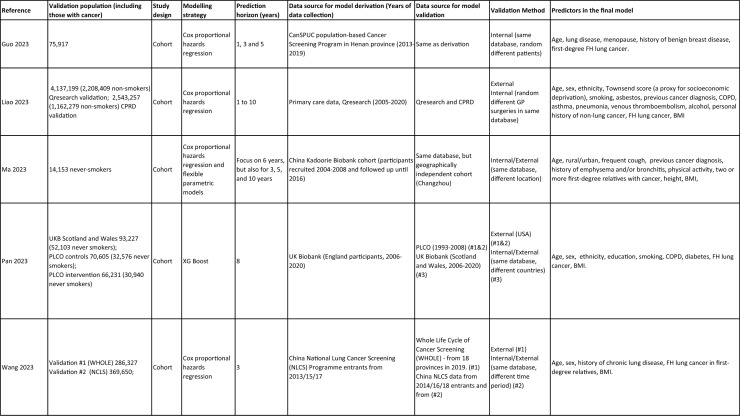

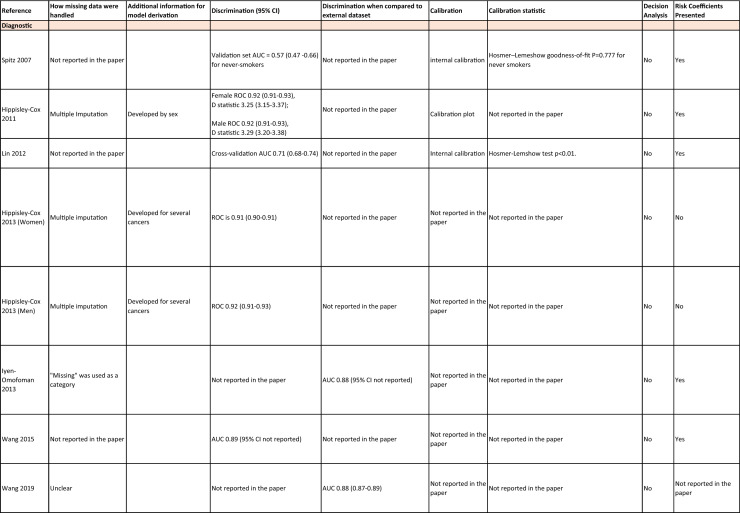

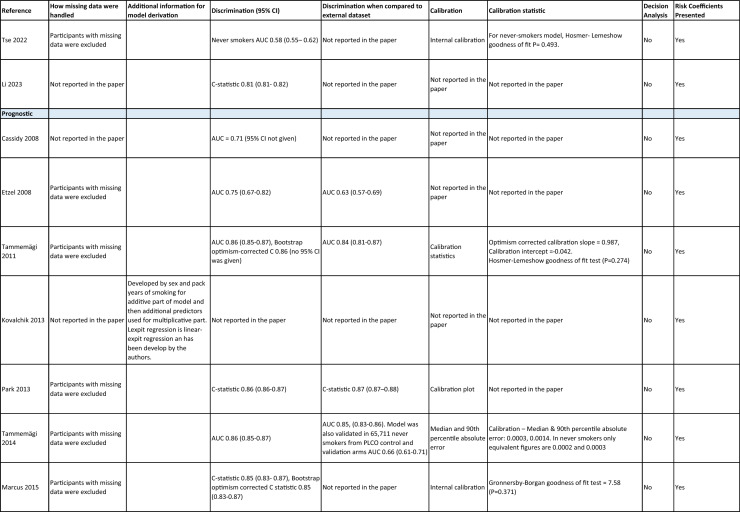

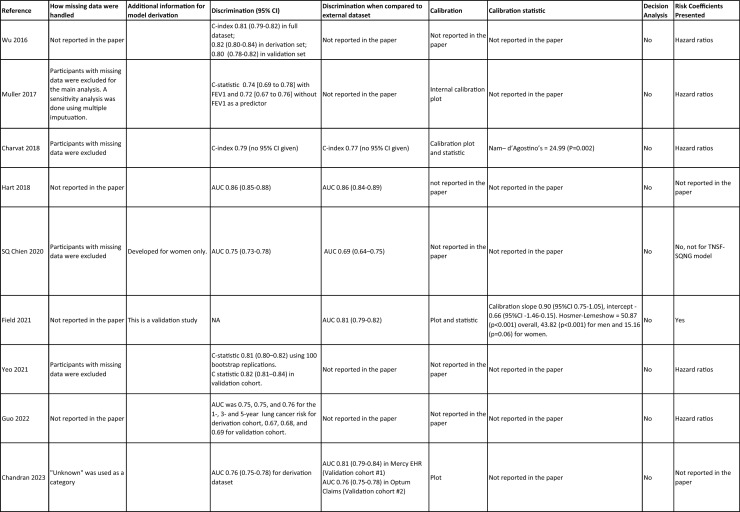

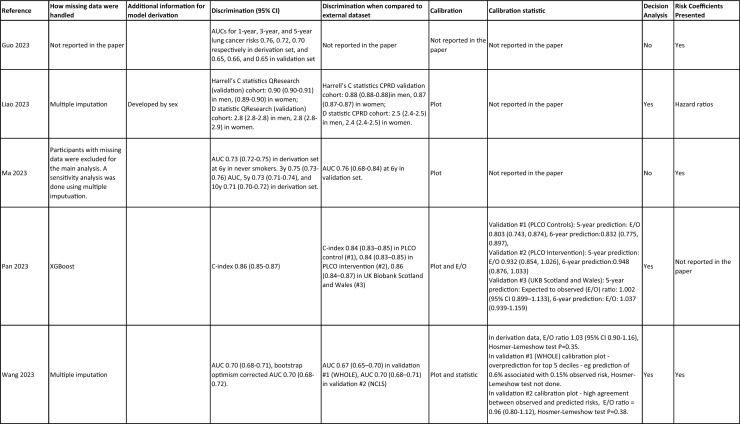
Summary of information from the included studies

### Risk of bias assessment

Two researchers independently used the Prediction model Risk Of Bias ASsessment Tool (PROBAST) [44] to assess risk of bias in four methodological domains of the included models: participants, predictors, outcome, and statistical analysis. Possible answers to signaling questions within each domain included “yes,” “probably yes,” “probably no,” “no,” and “unclear.” Answers to signaling questions were used in combination with the reviewers’ judgement, to assess the level of risk in each domain (low, high, or uncertain risk). If reviewers’ decisions differed, a third researcher reviewed and resolved the conflicts, discussing with the two reviewers when necessary.

## Results

Among 2,431 studies reviewed and screened, we found 31 models suitable for use in the primary care setting or at a population level, with study populations aged 18–89 years old and study data from 1991 to 2023. Eight models were derived exclusively from never-smokers [32, 45, 51], and 23 models from the general population (including both ever- and never-smokers) [10, 52, 73]. Twenty-five models were for both sexes, while three models were developed exclusively for men [50, 58, 62] and three for women [47, 51, 59]. Countries and territories of origin included the UK (10 studies) [10, 49, 53, 55, 58, 60, 64, 72, 73] China [8] [32, 46, 48, 50, 57, 65, 71], the US [7] [52, 54, 56, 63, 67, 68, 70], Korea [2] [62, 69], Taiwan [2] [45, 51], Italy [1, 61], and Japan [1, 66]. Ten were diagnostic models [50, 52, 55, 57, 60, 65, 68, 71], some including clinical features of lung cancer as predictors, which aimed to expedite diagnosis of existing lung cancer within a 1–2-year horizon. The remaining 21 studies were prognostic models (models to predict the likelihood/risk for future diagnosis of lung cancer), with prediction horizons ranging from > 2 to 10 years [10, 32, 45, 49, 51, 53, 54, 56, 61, 64, 66, 67, 69, 70, 72, 73].

One diagnostic [50] and seven prognostic [32, 45, 49, 51] models were derived exclusively from never-smokers in China [5] [32, 46, 48, 50], Taiwan [2], [45, 51] and the UK [1] [49]. Six were cohort studies [32, 45, 49], using Cox proportional hazards to develop models. The smallest cohort consisted of 75,917 never-smokers, and 119 developed lung cancer [47]; while the largest cohort, the China National Cancer Centre Lung Cancer model (NCC-LC_m2021_), included 425,626 never-smokers, of whom 2269 developed lung cancer [32]. The two case–control studies [50, 51] used logistic regression with 132–1511 cases and 536–5852 controls. For studies with a case–control design and using logistic regression, age- and sex-specific lung cancer incidence rates are needed to calculate absolute risk.

Of the 23 models developed from the general population (including ever- and never-smokers), 14 were derived from cohort studies [55, 56, 58, 59, 62, 64, 66, 70, 72, 73], with the smallest cohort using 8,760 people, of whom 237 developed lung cancer [64], while the largest, CanPredict (Lung), used 12,991,042 English primary care patients, of whom 73,380 developed lung cancer [72]. This model was built by updating a previous model [74] adding four predictors (family history of lung cancer, pneumonia, venous thrombo-embolism, and body mass index) and providing a flexible prediction horizon of 1–10 years [72]. Cox proportional hazards were used to analyze six population-based cohort studies [55, 62, 64, 66, 69, 72], while others used machine learning methods such as least absolute shrinkage and selection operator (LASSO) [70], XGBoost [68, 73], neural networks [67], and logistic [56, 63] and multinomial [58, 59] logistic regressions. Eight population-based case–control studies [52, 54, 57, 60, 61, 65, 71] and an external validation of a modified LLP case–control model [10] were analyzed using logistic regression using 491–12,074 lung cancer cases and 497–120,731 controls [54, 60].

Five out of 31 studies (16%) used multiple imputation [32, 55, 58, 59, 72] and two (6%) used XG boost [68, 73] to deal with missing data. Two (6%) studies used multiple imputation as a sensitivity analysis, rather than as the main analysis [48, 49]. The other 22 (71%) studies excluded participants with missing data, or did not report how to deal with missing data, or used missing data as a separate category in their studies [10, 45, 47, 50, 54, 56, 57, 60, 67, 69, 71]. Other methodological issues included [1] using univariate analyses to select predictors (12 studies, 39%), which is not recommended by the TRIPOD statement; [2] categorization of continuous predictors such as age (10 studies, 32%), which is unnecessary and causes loss of information from continuous variables; and [3] incorrect use of logistic regression to analyze a cohort study.

We summarized seven domains of predictors from the published models for never-smokers, with predictors for each model summarized in Table 2. Firstly, demographics–age, sex, ethnicity, socioeconomic status (education, Townsend score, zip code, and income); secondly, symptoms and clinical features indicative of lung cancer–hemoptysis, dyspnea, loss of appetite, loss of weight, fatigue, chest/shoulder pain, voice hoarseness, finger clubbing, thrombocytosis, C-reactive protein, alpha-fetoprotein, and carcinoembryonic antigen, relevant to diagnostic models; thirdly, medical history and comorbidities–personal history of cancer, chronic obstructive pulmonary disease (COPD), tuberculosis (TB), pneumonia, upper respiratory tract infection (URTI), lower respiratory tract infection (LRTI), chest infection, asthma, hay fever, hypertension, heart disease, stroke, venous thrombo-embolism, diabetes; fourthly, family history–family history of lung cancer (may or may not specify first degree relative), or 2 + family members with cancer; fifthly, lifestyle factors—alcohol, dietary intake, physical activity; sixthly, environmental risk factors—passive smoking (hours of daily exposure to smoke), asbestos, dust, pesticide, pollutants, carcinogens, cooking fuel exposure, residential radon exposure, and mask use in workplace as a proxy indicator of inhalation risk, and finally, examination and investigation findings–height, body mass index (BMI), forced expiratory volume in one second (FEV1), chest X-ray having been requested, fibrinogen, hyperlipidemia, bilirubin, and plasma glucose level.

Twenty-nine studies (94%) reported validation. Six (19%) models were externally validated using independent databases [10, 32, 54, 70, 72, 73]. Six (19%) models used internal–external validation by using the same databases at different time periods [51, 60, 62, 66, 68], or in different locations [48]. Seventeen models (55%) were internally validated, using bootstrapping [45, 49, 64, 69, 71], tenfold cross-validation [50, 53, 57], randomly splitting the dataset into derivation and validation populations [46, 47, 52, 55, 58, 59, 67, 69], or using the intervention group of the randomized controlled trial (RCT) as the validation population [56, 63], where the control group was used for derivation. One model was not validated [61] and another was incorrectly validated on its unmodified derivation dataset [65]; 95% CIs for discrimination were not presented for three models [53, 60, 66]. For never-smoker models, AUCs for internal–external validation ranged from 0.67 (95% CI 0.65–0.70) [32] to 0.76 (95% CI 0.68–0.84) [48], and for internal validation from 0.58 (95% CI 0.55–0.62)[50] to 0.80 (95% CI 0.78–0.82) [45]. For models including ever- and never-smokers, the AUCs ranged from 0.63 (95% CI 0.57–0.69) [54] to 0.87 (95% CI 0.87–0.87) [72] for external validation, from 0.77 (no 95% CI)(66) to 0.88 (95% CI 0.87–0.89) [68] for internal/external validation, and from 0.57 (95% CI 0.47–0.66)(52) to 0.92 (95%CI 0.91–0.93) [55] for internal validation. Calibration plots were presented in 10 out of 31 studies: five (16%) were externally calibrated [10, 32, 70, 72, 73], three (10%) were internally/externally calibrated [48, 62, 66], and two (6%) internally calibrated [49, 55]. Four models (13%) were inappropriately calibrated in their derivation datasets [50, 52, 57, 64], rather than in the validation dataset. Three (10%) papers categorized risk predictions prior to assessing calibration [45, 47], an approach that is not recommended [75]. Fourteen (45%) studies did not mention calibration at all.

Reporting the whole regression formula, or coefficients or odds/hazard ratios for the predictors is essential for model updating, recalibration, and external validation by other researchers, as well as good research practice recommended by the EQUATOR Network (https://www.equator-network.org/); six (19%) studies did not fully report such essential information [58, 59, 67, 68, 70, 73]. Decision curve analysis was only presented in three models [32, 72, 73].

### Risk of bias assessment

**Fig. 2:**
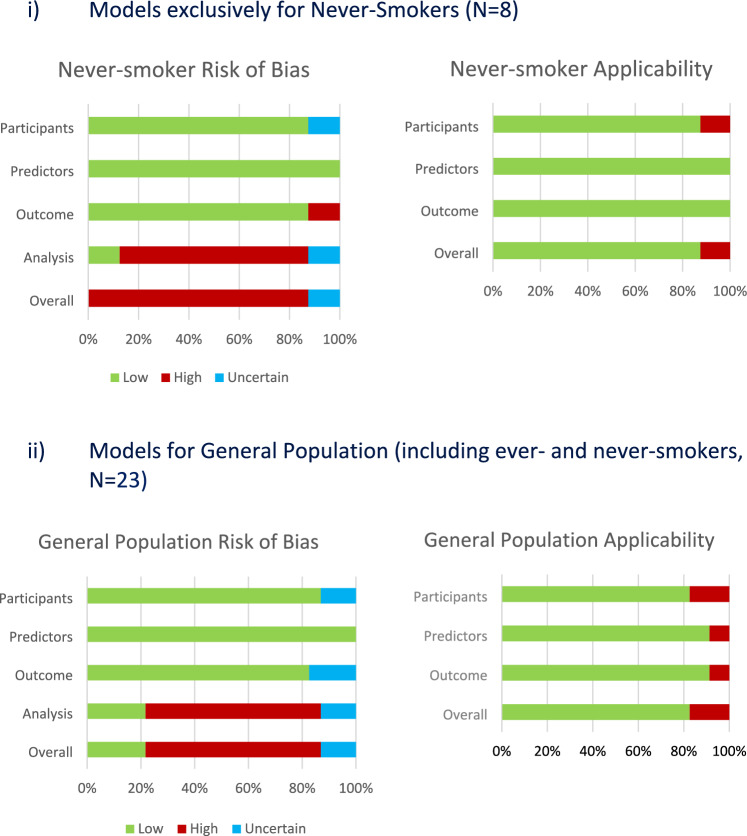
PROBAST (prediction of risk of bias assessment tool) Risk of Bias and Applicability for (i) Never-smoker Models and (ii) General Population (Ever-and Never-Smokers) Models

Twenty-six studies (84%), including all eight (100%) of the never-smoker models, were at high or uncertain risk of bias as shown in Fig. 2. Five (22%) models from the general population were at low risk of bias overall, and were all derived from cohort studies [55, 68, 70, 72, 73]; two were diagnostic models, QCancer (lung) [55] and Wang (2019) [68], predicting lung cancer diagnoses within 1–2 years by including patients presenting with potential symptoms, and the remaining three were prognostic models: Chandran [70], OWL [73], and CanPredict (lung) [72].

No study was at high risk of bias in the predictor domain. Four studies (13%) were at uncertain risk of bias in the participant domain, because they did not clearly report inclusion/exclusion criteria in the papers [32, 57, 61, 66]. Four studies (13%) were at high or uncertain risk of bias in the outcome domain due to a lack of evidence that the outcome was prespecified, determined in the same way for all participants or made without knowledge of the participants’ predictors [32, 58, 59, 66, 67]. Twenty-five studies (81%) were at high or uncertain risk of bias in the analysis domain, because of not dealing with missing data appropriately [10, 45, 54, 56, 57, 60, 67, 69, 71], incorrect selection methods for or categorization of predictors [46, 48, 50, 52, 54, 60, 62, 64, 65, 69, 71], or inadequate model evaluation [51, 53, 54, 60, 61, 65, 67] as mentioned above. Table 3.

**Table 3:** Assessment of risk of bias from the included studies

Study	Year	Model derived exclusively from never-smokers	Risk of bias				Applicability			Overall	
			Participants	Predictors	Outcome	Analysis	Participants	Predictors	Outcome	Risk of Bias	Applicability
Diagnostic (D)											
Spitz	2007	N	+	+	+	−	+	+	+	-	+
Hippisley-Cox	2011	N	+	+	+	+	+	+	+	+	+
Lin	2012	N	?	+	?	−	+	+	+	?	+
Hippisley-Cox (women)	2013	N	+	+	?	−	+	−	?	−	−
Hippisley-Cox (men)	2013	N	+	+	?	−	+	−	?	−	−
Iyen-Omofoman	2013	N	+	+	+	−	+	+	+	−	+
Wang	2015	N	+	+	+	−	+	+	+	−	+
Wang	2019	N	+	+	+	−	+	+	+	+	+
Tse	2022	Y	+	+	+	−	−	+	+	−	−
Li	2023	N	+	+	+	?	+	+	+	?	+
Prognostic (P)											
Cassidy	2008	N	+	+	+	−	+	+	+	−	+
Etzel	2008	N	+	+	+	−	−	+	+	−	−
Tammemägi	2011	N	+	+	+	−	+	+	+	−	+
Kovalchik	2013	N	?	+	+	−	+	+	+	−	+
Park	2013	N	+	+	+	−	−	+	+	−	−
Tammemägi	2014	N	+	+	+	−	+	+	+	−	+
Marcus	2015	N	+	+	+	−	+	+	+	−	+
Wu	2016	Y	+	+	+	−	+	−	+	−	−
Muller	2017	Y	+	+	+	−	+	+	+	−	+
Charvat	2018	N	?	+	+	?	+	+	+	−	+
Hart	2018	N	+	+	?	−	+	+	+	−	+
Chien	2020	Y	+	+	+	−	−	+	+	−	−
Field	2021	N	+	+	+	−	+	+	+	−	+
Yeo	2021	N	+	+	+	?	+	+	+	?	+
Guo	2022	Y	+	+	+	?	+	+	+	?	+
Chandran	2023	N	+	+	+	+	+	+	+	+	+
Guo	2023	Y	+	+	+	−	−	+	+	−	−
Liao	2023	N	+	+	+	+	+	+	+	+	+
Ma	2023	Y	+	+	+	−	+	+	+	−	+
Pan	2023	N	+	+	+	+	+	+	+	+	+
Wang	2023	Y	?	+	-	+	+	+	+	−	+

+ means at low risk of bias,−means at high risk of bias, ? means uncertain risk of bias

### Applicability

Five models (16%) were developed for specific populations, such as men, women, or Black American people, and would be applicable only to those specific populations. Two (6%) studies developed multinomial regression models predicting nine common male cancers [58], and eleven female cancers [59], in which lung cancer was one of these cancers. Some predictors in those two models were not specific to lung cancer. The remaining 23 studies (74%) were broadly applicable at the population level. However, the 9 models (29%), including China NCC-LC_m2021_ and OWL [73], derived from screening volunteers who may be more health-conscious than the general population, potentially suffered from self-selection/volunteer bias. Chandran’s model was derived from people with an outpatient visit in the past year [70], which might omit people who rarely consult a physician and potentially suffer from selection bias.

## Discussion

Among the 2,431 records retrieved from the four databases, after title and abstract screening and full-text review, 31 studies were included in this systematic review for critical appraisal and risk of bias assessment. Twenty-six studies (84%, 26/31) were at high risk of bias, with the majority (*n* = 23, 74%) not managing missing data correctly. Only five (16%) prediction models were at low risk of bias. Two were diagnostic models with prediction horizons of 1–2 years: QCancer (lung) [55] and Wang (2019) [68], the other three were prognostic models: Chandran [70], OWL [73], and CanPredict (lung) [72]. Chandran used a prediction horizon of three years [70], OWL eight years [73] and CanPredict (lung) offered more flexible prediction horizons, ranging from 1 to 10 years [72]. There is currently no model at low risk of bias predicting lung cancer exclusively for never-smokers, which is a research opportunity for future studies. The preferred study design for developing and validating a risk prediction model is a prospective longitudinal cohort study. However, for efficiency or costs, sampling patients using case–cohort and nested case–control designs can be applied [76].

To the best of our knowledge, this is the first systematic review of prediction models for lung cancer in never-smokers, giving an overview of all relevant studies published before 22 January 2025. We followed the most recent PRISMA statement [40] to conduct this study, used the CHARMS checklist [41] for data extraction and critical appraisal, presented a detailed description of the characteristics of the included models, used the TRIPOD statement [43] for model evaluation, and used the PROBAST tool [44] to assess risk of bias and applicability. All of these are good research practices and the strengths of this study. The critical appraisal of current literature and the research evidence summarized in this paper will be a valuable source of information for future studies.

We focused on prediction models applicable in primary care or the general population. A meta-analysis was not conducted, as our aim was to identify, summarize, and critique existing prediction models for lung cancer in never-smokers. A limitation of this study is that models involving pulmonary nodules found on imaging, or genetic biomarkers, were not included in this review, as radiology and genetics are specialized services, not widely accessible at the population level in some health systems. However, risk prediction models including genetic and radiological parameters would be helpful for further investigation of those identified at high risk of lung cancer from the population. A stepwise approach by risk stratification of the population first, followed by targeted investigations for those identified at high risk, may benefit patients, make good use of health resources, reduce the burden of the health system, and potentially avoid overdiagnosis and overtreatment by screening and treating individuals at low risk. It requires a thorough statistical and economic evaluation to establish a threshold for a model to classify people at low or high risk for subsequent interventions. Decision makers need to compare the discrimination, calibration, and net benefits among a series of models and select the best model(s) for their populations, and then evaluate the sensitivity, specificity, positive, and negative predictive values for different thresholds, the incidence, and disease burden of lung cancer in never-smokers in their populations, and the cost and human resources for those high-risk individuals to receive intervention after establishing the threshold.

Our results highlight the predominance of internally validated models with substantial variation in predictors and methodologies across populations. The findings underscore the need for transparent reporting, particularly the clear definition of the primary outcome, the handling of missing data, and the external validation [77]. The TRIPOD statement provides guidance for researchers conducting and transparently reporting prediction model studies. Most studies included in this review reported model discrimination, but calibration and decision curve analysis were reported less often, where calibration compares the predicted risks with observed frequencies of the outcome of a model, and decision curve analysis quantifies the net benefit of the model [75]. The interpretation of machine learning models is a concern. The interpretation of machine learning models is a concern. Some machine learning models are “black boxes” (uninterpretable), and are consequently impossible to externally validate or implement. If coefficients/weights for the predictors are known and reported, this may enable other researchers to validate the models in a different subset of the target population (generalizability) or different populations with different characteristics (transportability). Although not covered by this review, prediction models for prognosis (e.g., survival) for never-smokers diagnosed with lung cancer are also an important area for future research. A better understanding of prognosis can enable better-informed decision-making between the patient and physician, thus improving patient’s quality of life.

Prediction models for lung cancer are a very popular research topic. Three systematic reviews of the performance of lung cancer risk prediction models were conducted by different teams worldwide and published in 2025 [78, 80]. The first review by Juang and colleagues (Singapore) searched literature from PubMed and Embase up to January 2023, focusing on the model’s performance in Western and Asian populations [78]. They highlighted a significant research gap in prediction models for never-smokers, which is covered by our review. The second review by Zhang and colleagues (China) [79] searched literature from PubMed, Embase, and Web of Science up to 15 November 2023. They focused on two indicators for model performance: C-index (discrimination) and E:O ratio (calibration). The third review by Khalife and colleagues (Finland) [80] only searched literature from Medline (PubMed) up to March 2024 and aimed to evaluate the performance of externally validated risk prediction models and their potential applicability in lung cancer screening. We agree with their points that differences in population demographics and healthcare systems may limit the generalizability of models, and models should be optimized for local contexts to improve cost effectiveness in targeted screening programmes. Compared with the other three reviews, our review has a specific focus on the never-smoked population. We all reached a similar conclusion that the lack of external validation is a major issue for most published models.

Lung cancer in never-smokers differs in age, sex, histology, and mutational patterns from lung cancer in smokers, and weighting of its predictors may also differ [38]. Current high-quality RCT evidence based on smoking-related lung cancer screening may not directly transfer to never-smokers. Similarly, research evidence from East Asia [30, 32] may not be directly applicable to Western countries due to genetic susceptibility among different ethnicities, lifestyles, and environments. For example, cooking fumes are a risk factor for never-smoking East Asian women, but this is not applicable to Western lifestyles. Never-smokers are ineligible for lung screening programmes in Western countries irrespective of their lung cancer risk [6, 8, 81, 82], as the risk and benefit for lung cancer screening in never-smokers have not been established. However, never-smokers can be at high risk of developing lung cancer. For example, a 75-year-old male never-smoker with a past history of pneumonia, previous cancer history (not lung cancer), and a family history of lung cancer in a first-degree relative < 60 years has a 5-year risk for lung cancer of 5.5% based on the LLP_v2_ model (online calculator https://secure2.1s4h.co.uk/MYLUNGRISK/welcome.aspx). The design and optimization of care pathways for never-smokers at high risk are vital but are currently lacking. New care pathways need to be developed to enable proactive surveillance, early detection and medical intervention for never-smokers at high risk of lung cancer, involving all stakeholders including patients, carers, healthcare professionals, the public, and relevant charities, to coproduce new knowledge. Some charities in the UK have been dedicated to this population and this research area, and aim to increase public awareness. However, lung cancer in never-smokers is still a less investigated area with many research opportunities. We hope more researchers can work together to address the unmet needs of this population.

## Conclusion

Never-smokers can develop lung cancer, but this has been overlooked by both patients and physicians. We report major methodological issues in the current prediction models. Future research could use the findings from this study to develop and validate new prediction models, update existing models with additional predictors, adjust the weights of predictors to improve model performance, externally validate models in different populations to explore model generalizability, and further assess the clinical and cost effectiveness of the models for the intended population.

## Supplementary Information

Below is the link to the electronic supplementary material.

Supplementary file1 (PDF 1089 KB)

## Data Availability

No datasets were generated or analyzed during the current study.
