# Supplementary material for: Prediction models for early detection and diagnosis of lung cancer in people who have never smoked: a systematic review and critical appraisal
Source: Cancer Causes Control. 2026 May 19;37(6):88. doi: 10.1007/s10552-026-02173-w (PMC13186879; doi:10.1007/s10552-026-02173-w)
Supplement: Supplementary file 1 — Supplementary file1 (PDF 1089 KB) [file 10552_2026_2173_MOESM1_ESM.pdf]

Prediction models for early detection and diagnosis of lung cancer in never-smokers: a systematic review and critical appraisal

Authors

Judith Burchardt [1], Katherine Stokes [2], Yohance Victory [3], Weiqi Liao [4]

Institutions and affiliations

1. Department of Public Health and Policy, London School of Hygiene and Tropical Medicine, London, UK

2. Department of Psychology, University of Warwick, Coventry, UK

3. University of Bristol, Bristol, UK

4. Division of Cardiovascular Sciences, School of Medical Sciences, University of Leicester, Leicester, UK

Corresponding author

Dr Weiqi Liao, Email: weiqi.liao@leicester.ac.uk

Division of Cardiovascular Sciences, School of Medical Sciences, University of Leicester

Cardiovascular Sciences (Clinical Sciences Wing), Glenfield Hospital, Leicester, LE3 9QP

Supplementary materials

Table S1 – Search strategy

#	Searches in Ovid	Searches in EBSCO
**1**	"lung cancer"ab,hw,kf,kw,mh,ot,sh,ti,tw.	"lung cancer"
**2**	(lung adj3 cancer).ab,hw,kf,kw,mh,ot,sh,ti,tw.	(lung N3 cancer)
**3**	(lung adj3 neoplasm).ab,hw,kf,kw,mh,ot,sh,ti,tw.	(lung N3 neoplasm)
**4**	(lung adj3 carcinoma).ab,hw,kf,kw,mh,ot,sh,ti,tw.	(lung N3 carcinoma)
**5**	(lung adj3 tumor).ab,hw,kf,kw,mh,ot,sh,ti,tw.	(lung N3 tumor)
**6**	(lung adj3 tumour).ab,hw,kf,kw,mh,ot,sh,ti,tw.	(lung N3 tumour)
**7**	(lung adj3 malignan*).ab,hw,kf,kw,mh,ot,sh,ti,tw.	(lung N3 malignan*)
**8**	NSCLC.ab,hw,kf,kw,mh,ot,sh,ti,tw.	NSCLC
**9**	SCLC.ab,hw,kf,kw,mh,ot,sh,ti,tw.	SCLC
**10**	1 or 2 or 3 or 4 or 5 or 6 or 7 or 8 or 9	1 or 2 or 3 or 4 or 5 or 6 or 7 or 8 or 9
**11**	(no* adj3 smok*).ab,hw,kf,kw,mh,ot,sh,ti,tw.	(no* N3 smok*)
**12**	(never adj3 smok*).ab,hw,kf,kw,mh,ot,sh,ti,tw.	(never N3 smok*)
**13**	"never smok* ".ab,hw,kf,kw,mh,ot,sh,ti,tw.	"never smok*"
**14**	"non smok* ".ab,hw,kf,kw,mh,ot,sh,ti,tw.	"non smok*"
**15**	"nonsmok* ".ab,hw,kf,kw,mh,ot,sh,ti,tw.	"nonsmok*"
**16**	"non-smok* ".ab,hw,kf,kw,mh,ot,sh,ti,tw.	"non-smok*"
**17**	(passive adj3 smok*).ab,hw,kf,kw,mh,ot,sh,ti,tw.	(passive N3 smok*)
**18**	(second* adj3 smok*).ab,hw,kf,kw,mh,ot,sh,ti,tw.	(second* N3 smok*)
**19**	11 or 12 or 13 or 14 or 15 or 16 or 17 or 18	11 or 12 or 13 or 14 or 15 or 16 or 17 or 18
**20**	(predict* adj3 model).ab,hw,kf,kw,mh,ot,sh,ti,tw.	(predict* N3 model)
**21**	(predict* adj3 algorithm).ab,hw,kf,kw,mh,ot,sh,ti,tw.	(predict* N3 algorithm)

**22**	(predict* adj3 equation).ab,hw,kf,kw,mh,ot,sh,ti,tw.	(predict* N3 equation)
**23**	(predict* adj3 rule).ab,hw,kf,kw,mh,ot,sh,ti,tw.	(predict* N3 rule)
**24**	(predict* adj3 tool).ab,hw,kf,kw,mh,ot,sh,ti,tw.	(predict* N3 tool)
**25**	(risk adj3 predict).ab,hw,kf,kw,mh,ot,sh,ti,tw.	(risk N3 predict)
**26**	(risk adj3 model).ab,hw,kf,kw,mh,ot,sh,ti,tw.	(risk N3 model)
**27**	(risk adj3 assess).ab,hw,kf,kw,mh,ot,sh,ti,tw.	(risk N3 assess)
**28**	(risk adj3 tool).ab,hw,kf,kw,mh,ot,sh,ti,tw.	(risk N3 tool)
**29**	(risk adj3 score).ab,hw,kf,kw,mh,ot,sh,ti,tw.	(risk N3 score)
**30**	(risk adj3 stratif*).ab,hw,kf,kw,mh,ot,sh,ti,tw.	(risk N3 stratif*)
**31**	(decision adj3 aid).ab,hw,kf,kw,mh,ot,sh,ti,tw.	(decision N3 aid)
**32**	(decision adj3 tool).ab,hw,kf,kw,mh,ot,sh,ti,tw.	(decision N3 tool)
**33**	(decision adj3 support).ab,hw,kf,kw,mh,ot,sh,ti,tw.	(decision N3 support)
**34**	nomogram.ab,hw,kf,kw,mh,ot,sh,ti,tw.	nomogram
**35**	(predict* adj3 score).ab,hw,kf,kw,mh,ot,sh,ti,tw.	(predict* N3 score)
**36**	20 or 21 or 22 or 23 or 24 or 25 or 26 or 27 or 28 or 29 or 30 or 31 or 32 or 33 or 34 or 35	20 or 21 or 22 or 23 or 24 or 25 or 26 or 27 or 28 or 29 or 30 or 31 or 32 or 33 or 34 or 35
**37**	10 and 19 and 36	10 and 19 and 36
**38**	remove duplicates from 37	remove duplicates from 37
**Total**	1,526	114

Notes:

1. Medline, EmBase, and PsycInfo databases were integrated into the Ovid platform. The terms were searched in the following fields in Ovid: ab (Abstract), hw (Heading Word), kf (Keyword Heading Word), kw (Keyword Heading), mh (MeSH), ot (Original Title), sh (Subject Headings), ti (Title), and tw (Text Word).
2. CINAHL was in EBSCO.

Table S2 – The PRISMA Checklist

Section and Topic	Item #	Checklist item	Location where item is reported
**TITLE**			
Title	1	Identify the report as a systematic review.	Title
**ABSTRACT**			
Abstract	2	See PRISMA 2020 for Abstracts checklist.	Abstract
**INTRODUCTION**			
Rationale	3	Describe the rationale for the review in the context of existing knowledge.	introduction
Objectives	4	Provide an explicit statement of the objective(s) or question(s) the review addresses.	First sentence of the last paragraph in the introduction
**METHODS**			
Eligibility criteria	5	Specify the inclusion and exclusion criteria for the review and how studies were grouped for the syntheses.	Table 1<br>Methods, (eligibility criteria)
Information sources	6	Specify all databases, registers, websites, organisations, reference lists and other sources searched or consulted to identify studies. Specify the date when each source was last searched or consulted.	Literature Search
Search strategy	7	Present the full search strategies for all databases, registers and websites, including any filters and limits used.	Table S1, S2
Selection process	8	Specify the methods used to decide whether a study met the inclusion criteria of the review, including how many reviewers screened each record and each report retrieved, whether they worked independently, and if applicable, details of automation tools used in the process.	“Screening” subsection.
Data collection process	9	Specify the methods used to collect data from reports, including how many reviewers collected data from each report, whether they worked independently, any processes for obtaining or confirming data from study investigators, and if applicable, details of automation tools used in the process.	“Data extraction” subsection + Author’s contribution
Data items	10a	List and define all outcomes for which data were sought. Specify whether all results that were compatible with each outcome domain in each study were sought (e.g. for all measures, time points, analyses), and if not, the methods used to decide which results to collect.	“Data extraction” subsection
	10b	List and define all other variables for which data were sought (e.g. participant and intervention characteristics, funding sources). Describe any assumptions made about any missing or unclear information.	“Data extraction” subsection
Study risk of bias assessment	11	Specify the methods used to assess risk of bias in the included studies, including details of the tool(s) used, how many reviewers assessed each study and whether they worked independently, and if applicable, details of automation tools used in the process.	Risk of bias” subsection
Effect measures	12	Specify for each outcome the effect measure(s) (e.g. risk ratio, mean difference) used in the synthesis or presentation of results.	NA
Synthesis methods	13a	Describe the processes used to decide which studies were eligible for each synthesis (e.g. tabulating the study intervention characteristics and comparing against the planned	Table 1 PICOTS

Section and Topic	Item #	Checklist item	Location where item is reported
		groups for each synthesis (item #5)).	
	13b	Describe any methods required to prepare the data for presentation or synthesis, such as handling of missing summary statistics, or data conversions.	NA
	13c	Describe any methods used to tabulate or visually display results of individual studies and syntheses.	NA
	13d	Describe any methods used to synthesize results and provide a rationale for the choice(s). If meta-analysis was performed, describe the model(s), method(s) to identify the presence and extent of statistical heterogeneity, and software package(s) used.	NA
	13e	Describe any methods used to explore possible causes of heterogeneity among study results (e.g. subgroup analysis, meta-regression).	NA
	13f	Describe any sensitivity analyses conducted to assess robustness of the synthesized results.	NA
Reporting bias assessment	14	Describe any methods used to assess risk of bias due to missing results in a synthesis (arising from reporting biases).	NA
Certainty assessment	15	Describe any methods used to assess certainty (or confidence) in the body of evidence for an outcome.	NA
**RESULTS**			
Study selection	16a	Describe the results of the search and selection process, from the number of records identified in the search to the number of studies included in the review, ideally using a flow diagram.	Figure 1 – The PRISMA diagram
	16b	Cite studies that might appear to meet the inclusion criteria, but which were excluded, and explain why they were excluded.	NA
Study characteristics	17	Cite each included study and present its characteristics.	Table 2
Risk of bias in studies	18	Present assessments of risk of bias for each included study.	Table 3, Figure 2
Results of individual studies	19	For all outcomes, present, for each study: (a) summary statistics for each group (where appropriate) and (b) an effect estimate and its precision (e.g. confidence/credible interval), ideally using structured tables or plots.	NA
Results of syntheses	20a	For each synthesis, briefly summarise the characteristics and risk of bias among contributing studies.	Table 2
	20b	Present results of all statistical syntheses conducted. If meta-analysis was done, present for each the summary estimate and its precision (e.g. confidence/credible interval) and measures of statistical heterogeneity. If comparing groups, describe the direction of the effect.	NA
	20c	Present results of all investigations of possible causes of heterogeneity among study results.	NA
	20d	Present results of all sensitivity analyses conducted to assess the robustness of the synthesized results.	NA
Reporting biases	21	Present assessments of risk of bias due to missing results (arising from reporting biases) for each synthesis assessed.	Table 3
Certainty of evidence	22	Present assessments of certainty (or confidence) in the body of evidence for each outcome assessed.	NA
**DISCUSSION**			
Discussion	23a	Provide a general interpretation of the results in the context of other evidence.	First paragraph in the “Discussion” and all of

Section and Topic	Item #	Checklist item	Location where item is reported
			“Research in Context” subsection
	23b	Discuss any limitations of the evidence included in the review.	Last paragraph “Research in context”
	23c	Discuss any limitations of the review processes used.	“Strengths and limitations” – third paragraph
	23d	Discuss implications of the results for practice, policy, and future research.	“Research in Context” subsection and Conclusion
**OTHER INFORMATION**			
Registration and protocol	24a	Provide registration information for the review, including register name and registration number, or state that the review was not registered.	Methods – first paragraph
	24b	Indicate where the review protocol can be accessed, or state that a protocol was not prepared.	Methods – first paragraph
	24c	Describe and explain any amendments to information provided at registration or in the protocol.	NA
Support	25	Describe sources of financial or non-financial support for the review, and the role of the funders or sponsors in the review.	Funding
Competing interests	26	Declare any competing interests of review authors.	Conflicts of Interest
Availability of data, code and other materials	27	Report which of the following are publicly available and where they can be found: template data collection forms; data extracted from included studies; data used for all analyses; analytic code; any other materials used in the review.	NA

From: Page MJ, McKenzie JE, Bossuyt PM, Boutron I, Hoffmann TC, Mulrow CD, et al. The PRISMA 2020 statement: an updated guideline for reporting systematic reviews. BMJ 2021;372:n71. doi: 10.1136/bmj.n71. This work is licensed under CC BY 4.0. To view a copy of this license, visit https://creativecommons.org/licenses/by/4.0/

		Domain 1: Participants				Domain 2: Predictors				
**Study**	**Model derived exclusively from never-smokers**	1.1 Were appropriate data sources used, e.g. cohort, RCT or nested case-control study data?	1.2 Were all inclusions and exclusions of participants appropriate?	**Domain-level RoB judgement**	**Domain-level applicability judgement**	2.1 Were predictors defined and assessed in a similar way for all participants?	2.2 Were predictor assessment made without knowledge of outcome data?	2.3 Are all predictors available at the time the model is intended to be used?	**Domain-level RoB judgement**	**Domain-level applicability judgement**
**Diagnostic**										
Spitz 2007	No	Yes	Probably Yes	Low	Low concerns	Yes	Unclear	Yes	Low	Low concerns
Hippisley-Cox 2011	No	Yes	Probably Yes	Low	Low concerns	Yes	Probably Yes	Yes	Low	Low concerns
Lin 2012	No	Unclear	Unclear	Unclear	Low concerns	Yes	Probably Yes	Yes	Low	Low concerns
Hippisley-Cox 2013 (women)	No	Yes	Probably Yes	Low	Low concerns	Yes	Probably Yes	Yes	Low	High concerns
Hippisley-Cox 2013 (Men)	No	Yes	Probably Yes	Low	Low concerns	Yes	Probably Yes	Yes	Low	High concerns
Iyen-Omofoman 2013	No	Probably Yes	Probably Yes	Low	Low concerns	Probably Yes	Yes	Yes	Low	Low concerns
Wang 2015	No	Yes	Probably Yes	Low	Low concerns	Yes	Probably Yes	Yes	Low	Low concerns
Wang 2019	No	Yes	Probably Yes	Low	Low concerns	Probably Yes	Probably Yes	Yes	Low	Low concerns
Tse 2022	Yes	Yes	Probably Yes	Low	High concerns	Yes	Yes	Probably No	Low	High concerns
Li 2023	No	Unclear	Probably Yes	Low	Low concerns	Yes	Probably Yes	Yes	Low	Low concerns
Prognostic										
Cassidy 2008	No	Yes	Probably Yes	Low	Low concerns	Yes	Unclear	Yes	Low	Low concerns
Etzel 2008	No	Probably Yes	Probably Yes	Low	High	Yes	Probably Yes	Probably Yes	Low	Low concerns
Tammemägi 2011	No	Yes	Probably Yes	Low	Low concerns	Yes	Probably Yes	Yes	Low	Low concerns
Kovalchik 2013	No	Yes	Unclear	Unclear	Low concerns	Yes	Probably Yes	Yes	Low	Low concerns
Park 2013	No	Yes	Probably Yes	Low	High concerns	Yes	Yes	Yes	Low	Low concerns
Tammemägi 2014	No	Yes	Probably Yes	Low	Low concerns	Yes	Yes	Yes	Low	Low concerns
Marcus 2015	No	Yes	Yes	Low	Low concerns	Yes	Yes	Yes	Low	Low concerns
Wu 2016	Yes	Yes	Probably Yes	Low	Low concerns	Yes	Probably Yes	Probably No	Low	High concerns
Muller 2017	Yes	Yes	Probably Yes	Low	Low concerns	Yes	Probably Yes	Yes	Low	Low concerns
Charvat 2018	No	Yes	Unclear	Unclear	Low concerns	Yes	Yes	Yes	Low	Low concerns
Hart 2018	No	Yes	Probably Yes	Low	Low concerns	Probably Yes	Probably Yes	Yes	Low	Low concerns
Chien 2020	Yes	Probably Yes	Probably Yes	Low	High concerns	Yes	Probably Yes	Yes	Low	Low concerns
Field 2021	No	Yes	Yes	Low	Low concerns	Probably Yes	Yes	Yes	Low	Low concerns
Yeo 2021	No	Yes	Yes	Low	Low concerns	Yes	Probably Yes	Yes	Low	Low concerns
Guo 2022	Yes	Yes	Yes	Low	Low concerns	Probably Yes	Probably Yes	Yes	Low	Low concerns
Chandran 2023	No	Yes	Probably Yes	Low	Low concerns	Probably Yes	Probably Yes	Yes	Low	Low concerns
Guo 2023	Yes	Yes	Probably Yes	Low	High concerns	Yes	Probably Yes	Yes	Low	Low concerns
Liao 2023	No	Yes	Yes	Low	Low concerns	Probably Yes	Probably Yes	Yes	Low	Low concerns
Ma 2023	Yes	Yes	Probably No	Low	Low concerns	Probably Yes	Probably Yes	Yes	Low	Low concerns
Pan 2023	No	Yes	Probably Yes	Low	Low concerns	Probably Yes	Yes	Yes	Low	Low concerns
Wang 2023	Yes	Yes	Unclear	Unclear	Low concerns	Yes	Probably Yes	Yes	Low	Low concerns

	Domain 3: Outcome									
Study	3.1 was the outcome determined appropriately?	3.2 Was a pre-specified or standard outcome definition used?	3.3 Were predictors excluded from the outcome definition?	3.4 Was the outcome defined and determined in a similar way for all participants?	3.5 Was the outcome determine without knowledge of predictor information?	3.6 Was the time interval between predictor assessment and outcome determination appropriate?	**Domain-level RoB judgement**	**Domain-level applicability judgement**	4.1 Were there a reasonable number of participants with the outcome?	4.2 Were continuous and categorical predictors handled appropriately?
**Diagnostic**										
Spitz 2007	Yes	Yes	Yes	Yes	Yes	Probably Yes	Low	Low concerns	Yes	Probably No
Hippisley-Cox 2011	Probably Yes	Probably Yes	Probably Yes	Probably No	Unclear	Yes	Low	Low concerns	Yes	Probably Yes
Lin 2012	Yes	Yes	Yes	Probably No	Probably Yes	Yes	Low	Low concerns	Yes	Yes
Hippisley-Cox 2013 (women)	Probably Yes	Probably Yes	Unclear	Unclear	Unclear	Yes	Unclear	Unclear concerns	Yes	Yes
Hippisley-Cox 2013 (Men)	Probably Yes	Probably Yes	Unclear	Unclear	Unclear	Yes	Unclear	Unclear concerns	Yes	Yes
Iyen-Omofoman 2013	Probably Yes	Probably Yes	Probably Yes	Probably Yes	Unclear	Yes	Low	Low concerns	Yes	No
Wang 2015	Yes	Probably Yes	Yes	Yes	Probably Yes	Yes	Low	Low concerns	Yes	Probably No
Wang 2019	Yes	Yes	Probably Yes	Probably Yes	Probably Yes	Yes	Low	Low concerns	Yes	Probably Yes
Tse 2022	Yes	Yes	Probably Yes	Yes	Probably Yes	Yes	Low	Low concerns	Yes	Probably Yes
Li 2023	Yes	Yes	Yes	Yes	Yes	Yes	Low	Low concerns	Yes	Probably No
Prognostic										
Cassidy 2008	Yes	Yes	Yes	Probably No	Yes	Yes	Low	Low concerns	Yes	Yes
Etzel 2008	Yes	Yes	Yes	Probably Yes	Yes	Yes	Low	Low concerns	Yes	Probably Yes
Tammemägi 2011	Yes	Yes	Yes	Yes	Probably Yes	Yes	Low	Low concerns	Yes	Yes
Kovalchik 2013	Yes	Yes	Yes	Probably Yes	Probably Yes	Yes	Low	Low concerns	Yes	Yes
Park 2013	Yes	Yes	Yes	Probably No	Unclear	Yes	Low	Low concerns	Yes	Yes
Tammemägi 2014	Yes	Yes	Yes	Yes	Unclear	Yes	Low	Low concerns	Yes	Yes
Marcus 2015	Yes	Yes	Yes	Probably No	Unclear	Yes	Low	Low concerns	Probably Yes	Yes
Wu 2016	Yes	Yes	Probably Yes	Probably Yes	Unclear	Yes	Low	Low concerns	Yes	Probably No
Muller 2017	Yes	Yes	Yes	Probably Yes	Unclear	Yes	Low	Low concerns	Yes	Probably Yes
Charvat 2018	Probably Yes	Yes	Probably Yes	Probably Yes	Unclear	Yes	Low	Low concerns	Yes	Yes
Hart 2018	Unclear	Unclear	Probably Yes	Unclear	Unclear	Yes	Unclear	Low concerns	Yes	Probably No
Chien 2020	Yes	Probably Yes	Probably Yes	Probably Yes	Unclear	Yes	Low	Low concerns	Yes	Probably Yes
Field 2021	Yes	Yes	Yes	Yes	Unclear	Yes	Low	Low concerns	Probably Yes	Yes
Yeo 2021	Yes	Yes	Probably Yes	Probably Yes	Probably Yes	Yes	Low	Low concerns	Yes	Probably No
Guo 2022	Yes	Yes	Yes	Yes	Yes	Yes	Low	Low concerns	Yes	Probably No
Chandran 2023	Yes	Yes	Probably Yes	Yes	Probably Yes	Yes	Low	Low concerns	Yes	Yes
Guo 2023	Yes	Yes	Yes	Yes	Yes	Yes	Low	Low concerns	Yes	Probably No
Liao 2023	Probably Yes	Probably Yes	Probably Yes	Probably No	Unclear	Yes	Low	Low concerns	Yes	Probably Yes
Ma 2023	Yes	Yes	Yes	Yes	Probably Yes	Yes	Low	Low concerns	Yes	Probably No
Pan 2023	Yes	Yes	Yes	Probably Yes	Probably Yes	Yes	Low	Low concerns	Yes	Probably Yes
Wang 2023	Probably Yes	Unclear	Probably Yes	Probably Yes	Probably No	Yes	High	Low concerns	Yes	Probably Yes

	Domain 4: Analysis								Overall assessment	
Study	4.3 Were all enrolled participants included in the analysis?	4.4. Were participants with missing data handled appropriately?	4.5 Was selection of predictors based on univariable analysis avoided?	4.6 Were complexities in the data (e.g., censoring, competing risks, sampling of control participants) accounted for appropriately?	4.7 Were relevant model performance measures evaluated appropriately?	4.8 Were model overfitting and optimism in model performance accounted for?	4.9 Do predictors and their assigned weights in the final model correspond to the results from the reported multivariable analysis?	**Domain-level RoB judgement**	**Overall judgement about RoB**	**Overall judgement about applicability**
**Diagnostic**										
Spitz 2007	No	Unclear	No	Probably Yes	Probably Yes	Probably No	Yes	High	High	Low concerns
Hippisley-Cox 2011	Probably Yes	Yes	No	Probably Yes	Yes	Probably Yes	Yes	Low	Low	Low concerns
Lin 2012	Probably Yes	Unclear	Yes	Unclear	Yes	Probably Yes	Yes	High	Unclear	Low concerns
Hippisley-Cox 2013 (women)	Probably Yes	Yes	Yes	Probably Yes	No	Probably Yes	No	High	High	High concerns
Hippisley-Cox 2013 (Men)	Probably Yes	Yes	Yes	Probably Yes	No	Probably Yes	No	High	High	High concerns
Iyen-Omofoman 2013	Probably Yes	Unclear	No	Unclear	No	Probably Yes	Yes	High	High	Low concerns
Wang 2015	Probably Yes	Unclear	Yes	Probably Yes	No	Probably No	Yes	High	High	Low concerns
Wang 2019	Probably Yes	Probably Yes	No	Probably Yes	No	Probably Yes	Unclear	Low	Low	Low concerns
Tse 2022	Probably Yes	Unclear	No	Probably Yes	Yes	Probably No	Yes	High	High	Low concerns
Li 2023	Probably Yes	Unclear	Unclear	Probably Yes	No	Probably Yes	Unclear	Unclear	Unclear	Low concerns
Prognostic										
Cassidy 2008	Probably Yes	Unclear	Probably No	Probably Yes	No	Probably Yes	Yes	High	Low	Low concerns
Etzel 2008	Probably Yes	No	No	Unclear	No	Probably Yes	Probably No	High	High	High concerns
Tammemägi 2011	No	No	Yes	Probably Yes	Yes	Yes	Yes	High	High	Low concerns
Kovalchik 2013	Probably Yes	Unclear	Unclear	Probably Yes	No	No	Yes	High	High	Low concerns
Park 2013	Probably Yes	Probably No	No	Probably Yes	Yes	Yes	Yes	High	High	High concerns
Tammemägi 2014	Unclear	Unclear	Yes	Probably Yes	Yes	Yes	Yes	High	High	Low concerns
Marcus 2015	Probably Yes	Probably No	No	Probably Yes	Probably Yes	Yes	No	High	High	Low concerns
Wu 2016	Probably Yes	Unclear	Yes	Unclear	Probably Yes	Probably Yes	Probably No	High	High	Low concerns
Muller 2017	Probably Yes	No	Yes	Unclear	Probably Yes	Probably Yes	Yes	High	High	Low concerns
Charvat 2018	Unclear	Unclear	Yes	Probably Yes	Yes	Probably Yes	Yes	Unclear	High	Low concerns
Hart 2018	Probably Yes	Unclear	Yes	Probably Yes	No	Probably Yes	Unclear	High	High	Low concerns
Chien 2020	Probably Yes	Probably No	Yes	Yes	No	Probably Yes	Yes	High	High	Low concerns
Field 2021	Yes	Unclear	Yes	Probably Yes	Yes	Probably Yes	Yes	High	High	Low concerns
Yeo 2021	Probably Yes	Unclear	Yes	Yes	No	Probably Yes	Yes	Unclear	Unclear	Low concerns
Guo 2022	Probably Yes	Unclear	No	Probably Yes	Probably Yes	Probably Yes	Yes	Unclear	Unclear	Low concerns
Chandran 2023	Probably Yes	Probably No	Yes	Probably Yes	Yes	Probably Yes	Unclear	Low	Low	Low concerns
Guo 2023	Probably Yes	Unclear	No	Probably Yes	No	Probably Yes	Yes	High	High	High concerns
Liao 2023	Probably Yes	Yes	Yes	Probably Yes	Yes	Yes	Yes	Low	Low	Low concerns
Ma 2023	No	No	No	Probably Yes	Yes	Probably Yes	Yes	High	High	Low concerns
Pan 2023	Probably Yes	Yes	Yes	Probably Yes	Yes	Probably Yes	Unclear	Low	Low	Low concerns
Wang 2023	Unclear	Yes	Yes	Probably Yes	Yes	Yes	Yes	Low	High	Low concerns
